# Editorial for Special Issue “New Insight: Enzymes as Targets for Drug Development in Current Issues in Molecular Biology”

**DOI:** 10.3390/cimb47110920

**Published:** 2025-11-05

**Authors:** Sung-Kun Kim

**Affiliations:** Department of Natural Sciences, Northeastern State University, Tahlequah, OK 74014, USA; kim03@nsuok.edu

We are pleased to introduce this Special Issue titled ‘New Insight: Enzymes as Targets for Drug Development in Current Issues in Molecular Biology,’ which highlights recent progress and updates in molecular and computational approaches to understanding disease mechanisms and discovering new therapeutics. Enzymes continue to play a vital role in modern drug discovery, acting as accessible targets in a wide range of therapeutic fields such as oncology, infectious diseases, metabolic conditions, and neurodegenerative disorders. Contemporary strategies focus on improved selectivity, validation of targets using structure-based methods, and the combined use of computational experimental technologies. This collection of articles, five original research papers and two reviews, shows the value of structure-based modeling, enzyme inhibition studies, and natural compound screening in elucidating biochemical pathways and developing novel treatments across a broad range of diseases. The contributing authors from multiple countries and disciplines reflect the truly global nature of current biomedical research.

Molecular biology has transformed our ability to explore disease at atomic and mechanistic levels. The combination of in silico modeling, supramolecular chemistry, and pharmacological testing now allows scientists to explain interconnected biological interactions and predict molecular interventions effectively. Collectively, these investigations highlight a central theme, where combining computational accuracy with experimental validation is accelerating the development of enzyme-focused drug discovery.

The promise of dynamic combinatorial chemistry, an approach that creates libraries of molecules capable of adapting to bind specific biological targets, is highlighted by Martynov and colleagues, who expanded classical phosphodiesterase (PDE) inhibitor scaffolds by synthesizing novel dipyridamole derivatives [1]. Using molecular dynamics and combinatorial acylation, the authors synthesized eighteen supramolecular derivatives and identified an optimized dynamic dipyridamole derivative (DDD) with an improved PDE inhibition with nanomolar potency. In a mouse model of streptomycin-induced colitis with cyclophosphamide-induced immunodeficiency, DDD demonstrated both antidiarrheal and immunorestorative effects. This study indeed highlights how dynamic combinatorial chemistry can expand the therapeutic scope of classical small molecules, giving us a foundation for next-generation immunomodulatory agents.

The study by Alharbi focuses on the SARS-CoV-2 nsp10–16 methyltransferase, an enzyme essential for viral RNA cap modification and immune evasion, showing computational structure-based drug discovery including virtual screening, molecular docking, and molecular dynamics simulations [2]. The author identified several promising inhibitors with excellent binding affinity. Key stabilizing residues in the enzyme were also shown to maintain the high-affinity interactions. These approaches demonstrate the efficiency of computational modeling in identifying viable antiviral leads with detailed information and underscore the potential of enzyme inhibition as a good strategy for future coronavirus therapeutics.

Enhancing selectivity with the inhibitor landscape is always important. Zhang et al. developed and validated a series of selective PAK4 kinase inhibitors [3], addressing kinase selectivity and anti-cancer innovation. The authors examined structural differences between PAK1 and PAK4, related to tumor progression, to enhance the selectivity. By combining cross-docking analysis, protein conformation ensemble screening, and deep learning-based rescoring using GNINA, the authors cleverly identified STOCK7S-56165 as a promising scaffold for selective PAK4 inhibition. Further optimization through fragment replacement guided by electrostatic surface matching led to Compound 26, exhibiting enhanced electrostatic complementarity and binding affinity. This study introduces a refined computational strategy for designing inhibitors with improved selectivity, offering valuable insight into the development of anti-cancer therapies.

The clinical relevance of metabolic and signaling enzymes has also been spotlighted in the recent literature. Malic enzyme 1 (ME1) connects its function in redox homeostasis to stemness, metastasis, and energy reprogramming in hypoxic, aggressive tumors [4,5]. Consequently, inhibition of ME1 emerges as a strategy to curb malignancy at its metabolic core. The inhibitory effects of extracts from Indonesian medicinal plants on pancreatic lipase were examined, revealing their utility as natural and safe obesity treatments—a promising example of enzyme inhibition for metabolic disease management [6,7]. Natural compounds are important enzyme modulators because they have diverse structures, have been refined by evolution, and are often less toxic. This article shows how these natural products can work well alongside synthetic approaches.

Innovations in signaling pathway manipulation have been reported addressing intrahepatic cholangiocarcinoma’s resistance mechanisms [8,9]. Their dual inhibition of the MAPK and PI3K pathways arrested cell proliferation in cancer models, though apoptosis was not induced, signaling the need for further optimization—such as combination regimens—when targeting these networks.

In the broader context, novel inhibitors of histone deacetylases (HDACs) were explored, illuminating enzyme regulation in epigenetic therapeutics. Their rational design and mechanistic exploration have substantial implications for both cancer and neurodegenerative diseases, exemplifying how enzyme modulation can reach beyond traditional boundaries [10,11,12].

A wide range of applications of computational and molecular biology tools have been used to address global health challenges. From dynamic supramolecular chemistry and molecular docking to pathway-specific cancer inhibition and natural enzyme modulators, the studies in this Special Issue are examples of the synergy between experimental and theoretical approaches. Together, these examples show a typical path in drug discovery: starting with broad experimental screening and moving toward targeted design using AI tools and structural databases.

As the field continues to evolve, such integrated disciplines will be instrumental in improving drug discovery with enhanced efficacy and selectivity. Upcoming directions in the field include using more multi-target inhibitors, applying machine learning to refine drug optimization, and using chemical biology methods to investigate new types of enzymes and how they are regulated. We aim to highlight the expanding frontiers of computational and experimental approaches. The articles in this Issue not only deepen our understanding of intricate biological systems but also demonstrate how interdisciplinary integration can translate molecular-level insights into meaningful therapeutic outcomes. We believe that the innovative methodologies and diverse perspectives shown here will spur continued progress in molecular medicine and drug discovery.
